# A Descriptive Analysis of Macronutrient, Fatty Acid Profile, and Some Immunomodulatory Nutrients in Standard and Disease-Specific Enteral Formulae in Europe

**DOI:** 10.3389/fnut.2022.877875

**Published:** 2022-05-10

**Authors:** Mar Ruperto, Ana Montero-Bravo, Teresa Partearroyo, Ana M. Puga, Gregorio Varela-Moreiras, Maria de Lourdes Samaniego-Vaesken

**Affiliations:** ^1^Department of Pharmaceutical and Health Sciences, Faculty of Pharmacy, Universidad San Pablo-CEU, CEU Universities, Madrid, Spain; ^2^Grupo USP-CEU de Excelencia “Nutrición para la vida (Nutrition for life)”, ref: E02/0720, Departamento de Ciencias Farmacéuticas y de la Salud, Facultad de Farmacia, Universidad San Pablo-CEU, CEU Universities, Boadilla del Monte, Spain

**Keywords:** enteral formulae, disease-specific formulae, fatty acid ratios, immune-modulatory formulae, macronutrients, standard formulae

## Abstract

Foods for special medical purposes (FSMPs) are commercially available formulations used as a source of nutrition when administered orally or by tube feeding. This study examines, for the first time, the nutritional composition of enteral formulae (EFs) according to European nutritional guidelines. We developed a descriptive study on 118 EFs from 2020 to 2021. Formulae were classified as standard (SFs) and disease-specific (DSF). According to the protein-energy content, SFs were classified into G1, normoprotein-normocaloric; G2, normoprotein-hypercaloric; G3, hyperproteic-normocaloric; and G4, hyperproteic-hypercaloric. Disease-related formulae for metabolic stress, renal, cancer, pulmonary, diabetes, malabsorption, and surgery were studied. Macronutrient distribution, fatty acid profile (monounsaturated [MUFA], polyunsaturated [PUFA], saturated [SFA]), derived fat quality indexes, and immuno-modulatory nutrients (omega-3, eicosapentaenoic acid [EPA], docosahexaenoic acid [DHA], arginine and nucleotides) per 1,500 kcal infused were calculated. In total, 53% were SFs, mainly normoproteic (G1, G2) with higher carbohydrate contents in normocaloric vs. hypercaloric SFs. The most balanced fatty acid profiles (MUFA: 17.7%; PUFA: 6.8%; SFA: 9.5%) belonged to G1. The PUFA/MUFA ratio: ≥0.5 was in 85.7% with a higher proportion of EPA+DHA (46%) vs. omega-3 (15.8%) in SFs. In DSFs (46.9%), higher carbohydrate content (>50%) was in malabsorption and surgery, whereas high-fat content (>50%) was in pulmonary and renal formulae. DSFs had higher SFA vs. MUFA content, except for diabetes. EPA and DHA were added in 45.5% (cancer, malabsorption, and surgery). Only 12.7% of DSFs had arginine and nucleotides. A higher proportion of SFs was found, in line with current European guidelines. Results highlighted a wide intra-group variability of nutrients among the formula selected. These findings are useful to evaluate the nutritional composition of EFs from a preventive and/or therapeutic perspective in clinical settings.

## Introduction

In total, 40% of hospitalized adult patients in the European Union (EU) suffer from disease-related malnutrition (DRM) with an estimated average cost to healthcare systems of 170 billion in Europe (1). An aging European population is associated with a significant increase in the prevalence of chronic communicable and non-communicable diseases (2), which partly justifies the rising incidence of DRM.

The clinical nutrition market is expected to experience a compound annual growth rate of 8.96% between 2021 and 2026 (3). Over the past few years, the expected exponential growth of nutritional products has been presumably associated with the incidence of chronic and metabolic disorders, the increasing preference for enteral over parenteral nutrition, and the rise of home enteral nutrition.

Medical nutrition therapy comprises oral nutritional supplements (ONS), enteral tube feeding (enteral nutrition, EN), and parenteral nutrition (4). EN by tube feeding is the first choice of artificial nutritional intervention in conditions of high nutritional risk or DRM, as long as the oral route does not meet nutritional needs, and the gastrointestinal tract is functional and permeable for intestinal absorption of nutrients. *Guidelines on Nutrition* (5, 6) from the European Society of Clinical Nutrition and Metabolism (ESPEN) recommends EN when the patient is expected to be unable to eat or if there is a compromised energy intake of estimated needs for at least 1 week (6). EN is more physiological, has fewer technical and infectious complications, and is also less expensive for healthcare systems (7, 8). Advances in commercial ready-to-use nutrition formulae and equipment for their delivery have made EN by tube feeding safe and efficacious to administer to adult patients in either hospital or home settings (5).

Foods for special medical purposes (FSMPs) are specially commercialized formulae that are administered *via* the digestive tube (orally or by tube feeding). They are intended for the partial or exclusive feeding of patients with a limited ability to ingest, digest, absorb, metabolize, or excrete certain nutrients contained therein, whose dietary management cannot be achieved by the usual diet. FSMPs are regulated under the framework of Directive 609/2013/EC of the European Parliament and the Council on foodstuffs intended for particular nutritional uses (PARNUTS) (9).

Adult enteral formulae are designed to provide the adult's recommended dietary allowances (RDA) when at least 1,500 kcal/day is administered (9). Globally, enteral formulae are usually classified into standard formulae (SFs) and disease-specific formulae (DSFs) whose composition meets the nutritional requirements for energy, protein, and micronutrients when administered as the sole source of nutrition (4, 10). SFs are nutritionally complete as they contain intact proteins (polymeric) frequently from casein and soy protein isolates. Energy density can range between 1.0 and 2.0 kcal/ml, providing a protein content of 15–25% and a lipid content of 30–50% of the total energy (TE) (6, 11, 12). The major energy source is provided by carbohydrates in the form of polysaccharides and glucose, while the lipid content comes mainly from long-chain triglycerides (LCT) and/or mixed with medium-chain triglycerides (MCT). Some formulae contain fiber and are lactose- and gluten-free.

Disease-specific enteral formulae (DSFs) are modified for addressing the demands of individual disease states that cannot be met by SFs. Both, SFs and DSFs called semi-elemental or peptide-based oligomeric formulae, are partially predigested and contain oligopeptides, short-chain peptides, glucose, oligosaccharides and MCT, and a low content in fat and a free-fiber content (6, 11, 12). Peptide-based oligomeric formulae are used for patients with impaired ability to digest or absorb intact nutrients (e.g., initial phase after prolonged fasting, selected patients with short bowel syndrome, enterocutaneous fistulas, and when administration is to jejunum in critical care or severe acute pancreatitis) (13).

Immuno-modulating formulations are enriched in some specific nutrients (e.g., omega-3, arginine, glutamine, and nucleotides) to achieve nutraceutical effects on the body's response to trauma, surgery, or infection (12).

Given the disease impact of FSMPs, specifically of the EFs administered by tube feeding, it is prescriptive to consider the macronutrient composition, fatty acid profile, and immuno-modulating substrates in order to achieve a tailor-made of enteral formulae in clinical settings. At present, there are no similar studies that analyse FSMPs from the perspective of nutritional composition in SFs and DSFs. Therefore, this study aimed to identify and describe some FSMPs currently administered by tube feeding at European level according to (a) the type of enteral formula (SFs and DSFs); (b) the macronutrients distribution; (c) the fatty acid profile and its fat quality ratios (to guide i.e., long-term cardiovascular disease [CVD] prevention); and (d) the content of immuno-modulatory nutrients with potential nutraceutical effect according to current European guidelines on clinical nutrition.

## Materials and Methods

### Study Design

Descriptive study of 118 FSMPs according to the European PARNUTS Directive (9) and marketed by eight clinical nutrition European laboratories was undertaken (Supplementary Annex 1).

Inclusion criteria: Nutritionally complete enteral formulae in liquid form for adults (≥18 years) for use and administration through nasoenteral tube or ostomy in accordance with FSMP's legislation, and legally permissible claims relating to the dietary treatment of a disease, disorder, or condition (required on a mandatory basis for FSMPs) were included.

Exclusion criteria: Infant and follow-on formulae, pediatric enteral formulae, free amino acid-formulae, and ONS in liquid, powder, or pudding forms that were not used as the sole source of nutrition were excluded.

### Method

Enteral formulae were classified according to the criteria previously established by Silk (10) into SFs and DSFs. Standard formulae were classified according to protein type (polymeric, peptide-based oligomeric, or semi-elemental) and protein content (normoproteic: ≤ 20% and hyperproteic: >20% protein of TE) as the main criteria. As a secondary criterion, SFs were classified according to energy density (normocaloric: 1.0–1.2 kcal/ml and hypercaloric: >1.2 kcal/ml of enteral formula). Overall, SFs were classified according to protein content and energy density into 4 groups (Gn): Group 1, normoproteic-normocaloric; Group 2, normoproteic-hypercaloric; Group 3, hyperproteic-normocaloric; and Group 4, hyperproteic-hypercaloric. For the DSFs, the therapeutic indication of each formulation was established as the primary criterion as follows: metabolic stress, renal, cancer, pulmonary and diabetes diseases, intestinal malabsorption, and surgery.

The data sheets of each enteral formula were examined, and the nutritional content per 100 ml was recorded from the information declared in the technical data sheets from vademecums and/or websites of each one of the clinical nutrition laboratories from 2020 to 2021. TE density (kcal), macronutrient distribution (proteins, carbohydrates, and lipids), fatty acid profile expressed in grams and percentage of total fat as monounsaturated fatty acids (MUFA), polyunsaturated fatty acids (PUFA), and saturated fatty acids (SFA) per 1,500 kcal were calculated. Clinical guidelines for the management of dyslipidemias and lipid modification to reduce CVD risk were used (14) with the following fatty acid profile distribution (MUFA: 20%, SFA: <7%, and PUFA: 5% of the TE). The PUFA/SFA ratio and the PUFA+MUFA-to-SFA ratio were calculated to assess the potential impact of these ratios on cardiovascular (CV) health (15, 16). A cut-off point for PUFA/SFA ratio ≥0.5 was set, while the cut-off point for PUFA+MUFA-to-SFA ratio was defined at a score ≥2.0 (both cut-off points were associated with low CVD risk) (16). Additionally, the origin of fat sources such as MCT, the content of omega-3 and/or omega-6, eicosapentaenoic acid (EPA), and docosahexaenoic acid (DHA) as well as l-carnitine, arginine, and nucleotides were also registered. The sum of EPA and DHA content (EPA+DHA) was analyzed in all those enteral formulae containing and/or declaring these components in the nutritional composition data sheet.

### Statistical Analysis

Data analyses were expressed as mean, percentage or both, depending on the variable analyzed, and the minimum and maximum (min-max) values of the interval for each one of the nutrients and fat quality ratios studied. No imputation was used to estimate missing data, and analyses were based on all available data reported. Statistical analysis was carried out using the Statistical Package for the Social Sciences (SPSS version 27) software.

## Results

### Global Data

**Figure 1:**
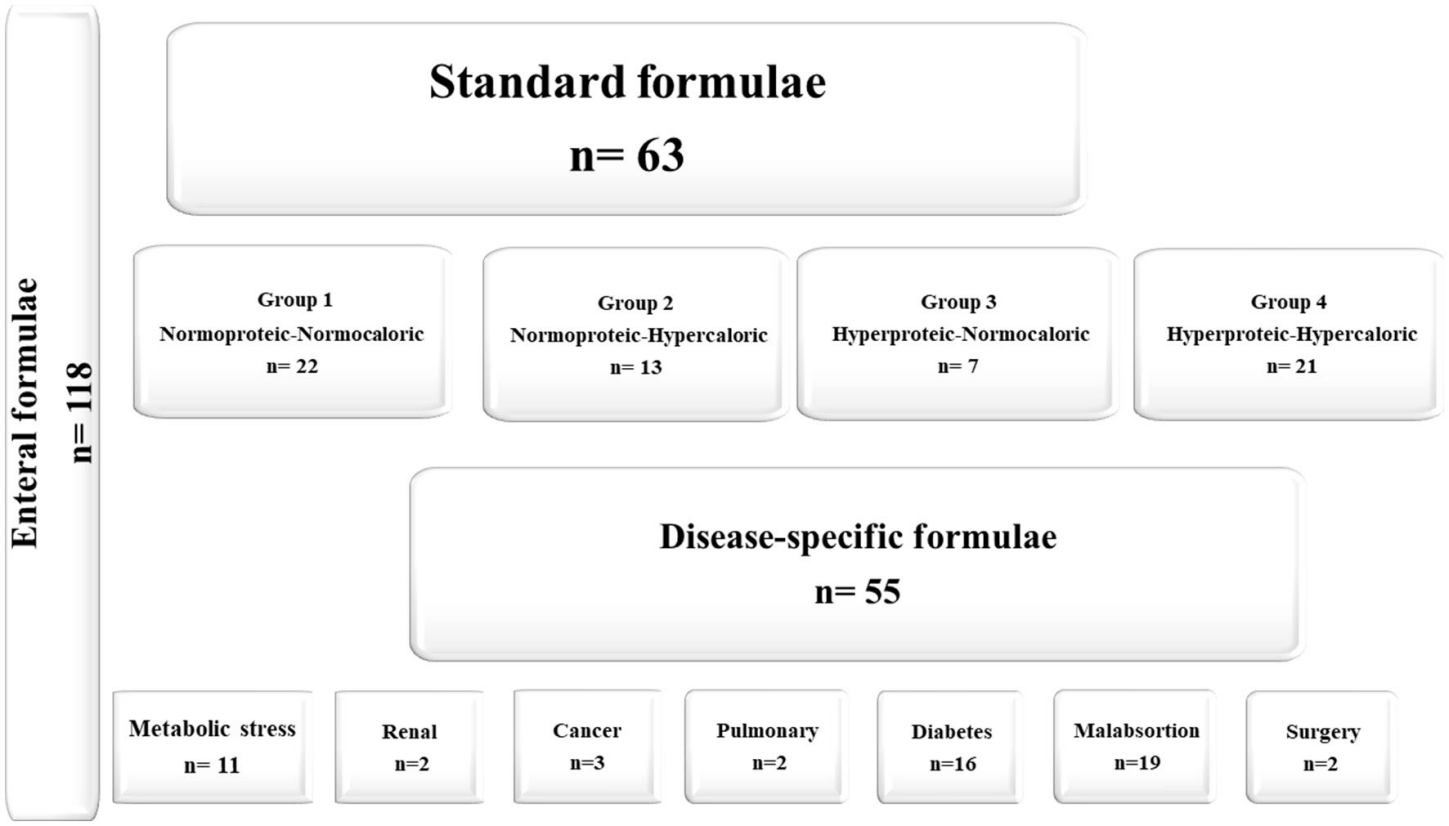
Characteristics of the enteral formulae evaluated in the study.

A total of 118 enteral formulae were studied from which 76.3% (*n* = 90) were marketed in the EU and 23.7% (*n* = 28) were marketed locally in Spain. SFs accounted for 53.4% (*n* = 63), all of which were polymeric formulae. DSFs accounted for 46.6% (*n* = 55). Figure 1 shows the classification of the SFs and DSFs.

### Descriptive Analysis of the Standard Enteral Formulae

Table 1 shows the macronutrient distribution, fatty acid profiles and their quality ratios, and some immunomodulatory nutrients in the FSMPs. Analyzing the frequency of SFs, normoproteic-normocaloric/hypercaloric formulae (G1, G2) accounted for 55.5% of the SFs, while a lower frequency of hyperproteic and normocaloric/hypercaloric formulae (44.5%) was found.

**Table 1:** Macronutrient distribution, fatty acid profile, fat quality ratios, and some immune-modulatory nutrients content in standard formulae^a^.

**Nutrients**	**Standard Formulae**			
	**Group 1. Standard Normoproteic-Normocaloric** ***n* = 22** ***Mean (min-max)***	**Group 2. Standard** **Normoproteic-Hypercaloric** ***n* = 13** ***Mean (min-max)***	**Group 3. Standard Hyperproteic-Normocaloric *n* = 7** ***Mean (min-max)***	**Group 4. Standard** **Hyperproteic-Hypercaloric** ***n* = 21** ***Mean (min-max)***
Water (mL)	1,238 (1,153.2–1,290.0)	702.9 (517.5–780.0)	1,206.8 (1,044.9–1,275.0)	836.6 (527.2–1,014.1)
Infused volume (mL)	1,451.3 (1,250.0–1,500.0)	944.5 (750.0–1000.0)	1,448.5 (1,282.0–1,500.0)	1,068.5 (750.0–1,239.7)
Energy (kcal/mL)^&^	1.03 (1.0–1.2)	1.60 (1.5–2.0)	1.03 (1.0–1.17)	1.44 (1.21–2.0)
Protein (g)	60.8 (56.1–82.5)	61.2 (56.0–68.0)	85.8 (75.0–96.0)	77.8 (63.0–119.0)
Carbohydrates (g)	190.9 (161.1–207.0)	180.9 (150.7–203.0)	186.6 (168.9–216.4)	162.5 (123.8–195.2)
Fiber (g)	10.9 (0.00–30.0)	5.16 (0.00–20.6)	11.2 (0.0–28.9)	8.05 (0.00–24.2)
Fat (g)	53.2 (46.0–60.0)	58.4 (47.8–75.0)	47.9 (39.0–53.4)	56.4 (43.4–75.0)
MCT (g)^#^	13.5 (4.5–31.5)	13.5 (8.8–26.0)	17.9 (7.2–30.0)	13.6 (5.9–33.0)
SFA, g	14.5 (4.5–40.5)	15.8 (5.0–29.0)	21.2 (10.5 −34.5)	16.3 (4.7–37.0)
MUFA, g	24.9 (10.2–34.5)	29.5 (10.0–43.5)	15.8 (4.3–28.9)	27.1 (5.0–42.8)
*PUFA*, g	13.6 (5.8–29.1)	11.3 (6.4–16.0)	9.2 (5.7–14.1)	11.6 (6.3–17.6)
PUFA/SFA ratio^†^	1.5 (0.2–4.0)	0.9 (0.3–3.2)	0.5 (0.2–0.7)	0.8 (0.4–2.5)
(PUFA+MUFA)/SFA^††^	4.3 (0.4–10.3)	3.3 (0.7–10.6)	1.6 (0.4–2.7)	3.0 (0.5–9.2)
Omega-3 (g)^*^	2.0 (1.5–2.8)	-	NA	5.9 (1.5–10.9)
Omega-6 (g)^**^	9.1 (3.7–13.6)	-	NA	8.7 (1.6–14.8)
EPA+DHA (mg)^***^	496.9 (436.9–688.1)	450.6 (330.4–656.3)	-	537.8 (390.0–608.5)
L-Carnitine (mg)^*^	134.4 (117.8–150)	136.4 (112.5–160.0)	140.0 (120.0–150.0)	114.5 (92.3–137.4)

a *Results are expressed as mean, minimum (min), and maximum (max) per 1,500 kcal of infused enteral formula. (-), only in one of the group's formulae; NA, not in formula*.

& *Energy content is expressed in kcal per mL of standard formula as mean, minimum, and maximum*.

*MCT, medium-chain triglycerides; MUFA, monounsaturated fatty acid; PUFA, polyunsaturated fatty acid; SFA, saturated fatty acid*.

# *Mean MCT content according to the different groups (Gn): G1 (n = 11 formulae), G2 (n = 5 formulae), G3 (n = 4 formulae), and G4 (n = 14 formulae)*.

† *PUFA/SFA ratio is composed of the polyunsaturated fatty acid (PUFA) content divided by the saturated fatty acid (SFA) content, both expressed in grams*.

†† *(PUFA+MUFA)/SFA ratio is the result of adding the content in grams of PUFA plus MUFA and dividing it by the content in grams of SFA*.

* Omega-3 and ^**^Omega 6 contents according to the different groups (Gn): G1 (n = 5 formulae), G2 (n = 1 formulae), and G4 (n = 4 formulae).

*** *EPA+DHA index is the sum of the eicosapentaenoic acid (EPA) content of the formula plus docosahexaenoic acid (DHA) expressed in mg per total volume of infused enteral formula. EPA+DHA mean content according to the groups (Gn): G1 (n = 8 formulae), G2 (n = 7 formulae), G3 (n = 1 formula), and G4 (n = 13 formulae)*.

^*^Mean carnitine content according to the different groups (Gn): G1 (n = 4 formulae), G2 (n = 6 formulae), G3 (n = 3 formulae), and G4 (n = 6 formulae).

**Figure 2:**
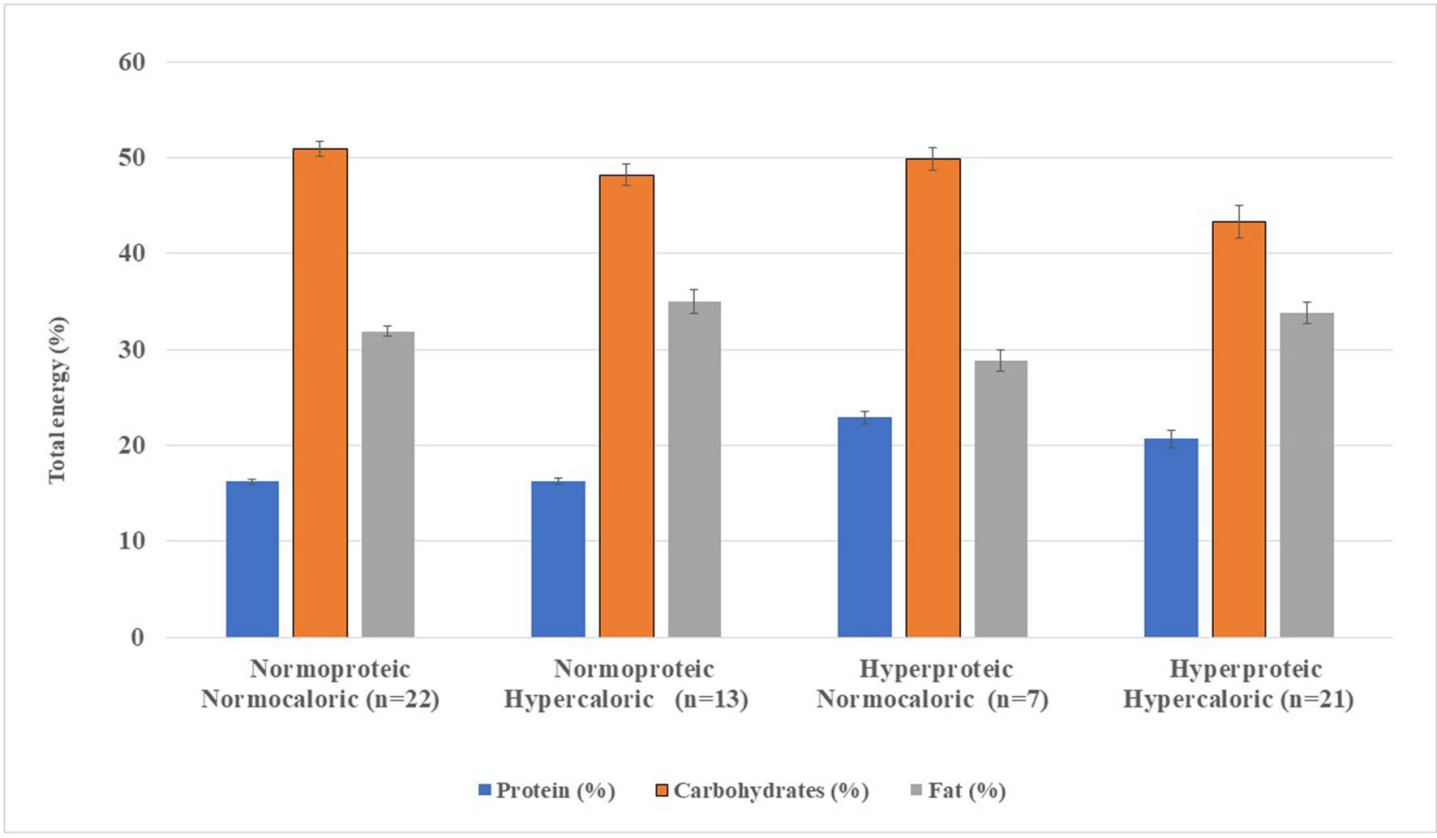
Macronutrient distribution from standard enteral formula per 1,500 kcal infused. Enteral formulae were classified according to protein content and energy density into four groups (Gn), namely, Group 1, normoproteic normocaloric; Group 2, normoproteic hypercaloric; Group 3, hyperproteic normocaloric; and Group 4, hyperproteic-hypercaloric standard formula. The error bars on each bar show the minimum and maximum value of the nutrient analyzed per 1,500 kcal of standard enteral formulae.

High-calorie SFs (G2, G4), regardless of protein content, provided a lower percentage of free-water in the range of 46.9–55.0%, while normocaloric SFs (G1, G3) ranged from 80.4 to 82.5% of free-water. The distribution of macronutrients expressed as a percentage of the mean TE of the SFs in 1,500 kcal is shown in Figure 2.

**Figure 3:**
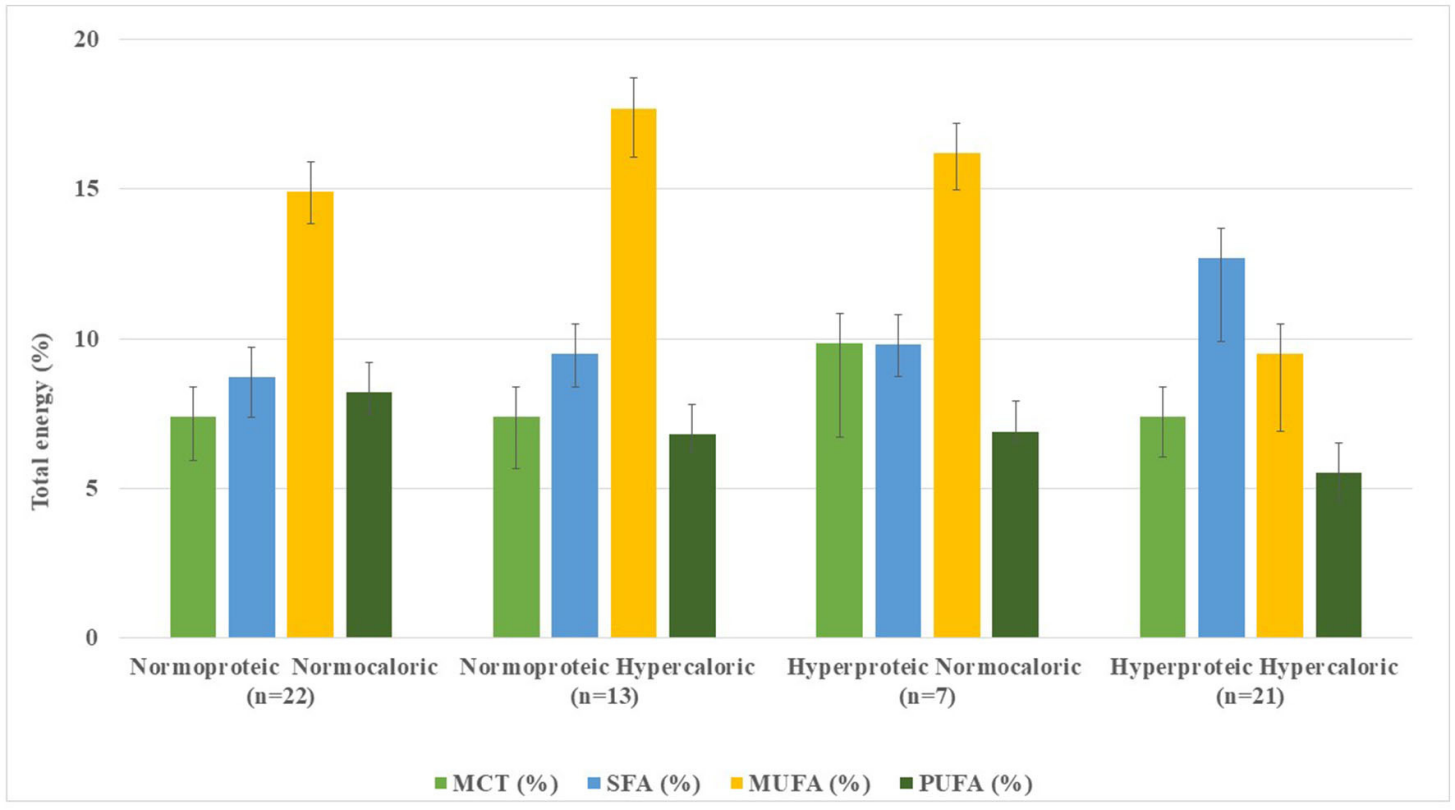
Distribution of fatty acids and medium-chain triglycerides content in standard enteral formulae per 1,500 kcal infused.

Protein content was higher in G3 and G4 (20.7–22.9%), with lower carbohydrate content (43.3%). Added fiber was found in 16 SFs (55%) in G1 and G2, while 46.4% in G3 and G4 were fiber-free (data not shown). Fat content was higher in G2 (35.0%; *n* = 13) compared with G4 (33.8%; *n* = 21) (Table 1, Figure 1). Medium-chain triglycerides (MCT) were in 53.9% (*n* = 34) of the SFs, with a mean MCT value of 13.5–17.9 g of TE. Fatty acid profiles and MCT expressed as a percentage of TE per 1,500 kcal are shown in Figure 3.

Of the 63 SFs, 7 formulae (11.3%) did not declare their fatty acid profiles in their data sheets. In 56 SFs, mean SFA content ranged from 8.7% (2.7–24.3%) of TE in G1 (*n* = 22) to 9.8% (2.8–22.2%) in G4 (*n* = 21); remarkably higher values were observed in G3 (*n* = 7) with 12.7% (6.3–20.7%) (Table 1, Figure 3). Conversely, MUFA content was higher in G2 (*n* = 13), with 17.7% (6.0–26.1%) of TE and lower in G3 (*n* = 7) accounting for 9.5% (2.6–17.3%) of TE. PUFA contents ranged from 5.5% (3.4–8.5%) of TE in G3 (*n* = 7) to 8.2% (3.5%−17.5.0%) of TE in G1 (*n* = 22) formulae. Mean fatty acid profile of G1 was the closest and most balanced (MUFA: 17.7%, SFA: 9.5%, and PUFA: 6.8% of TE) for guiding CVD prevention (14).

Of the SFs analyzed, only 85.7% (*n* = 48) had a PUFA/SFA ratio: ≥0.5, with mean ratio values of 1.20 (r: 0.51–4.00) (data not shown). Mean values of PUFA+MUFA-to-SFA ratio were 4.0 (r: 2.1–10.6), whilst in 11 formulae (19.6%) had a PUFA+MUFA-to-SFA ratio <2.0 (mean: 0.9; r: 0.4–1.9) (Table 1).

Omega-3 and omega-6 accounted for 15.8% of the SFs being mostly observed in G1 (8.0%) and G4 (6.3%). The EPA+DHA content was in 46% (*n* = 29), being only available in one of seven hyperproteic-normocaloric formulae (G3). l-carnitine was added to 19 SFs (r: 114.5–140.0). None of the SFs included immune-modulatory nutrients such as arginine and/or nucleotides in their nutritional composition.

### Descriptive Analysis of Disease-Specific Enteral Formulae

DSFs accounted for 46.6% (*n* = 55). Higher frequency was found for diabetes mellitus (16.6%; *n* = 16), malabsorption (16.1%; *n* = 19), and metabolic stress (9.3%; *n* = 11). Formulae used for kidney disease, cancer, or surgery only represented 5.1% (*n* = 7).

Polymeric DSFs accounted for 61.9% (*n* = 34) while oligomeric formulae were found in malabsorption (*n* = 13; 23.6%) and metabolic stress (*n* = 8; 14.5%). The macronutrient distribution, fatty acid profile, and fat quality ratios as well as the EPA+DHA contents are shown in Table 2.

**Table 2:** Macronutrient distribution, fatty acid profile, fat quality ratios, and some immunomodulatory nutrients in disease-specific formulae^a^.

**Nutrients**	**Disease-Specific Formulae**						
	**Metabolic stress *n* = 11** ***Mean (min-max)***	**Renal** ***n* = 2** ***Mean (min-max)***	**Cancer** ***n* = 3** ***Mean (min-max)***	**Pulmonary** ***n* = 2** ***Mean (min-max)***	**Diabetes** ***n* = 16** ***Mean (min-max)***	**Malabsorption** ***n* = 19** ***Mean (min-max)***	**Surgery** ***n* = 2** ***Mean (min-max)***
Water (mL)	986.2 (770–1,275.0)	574.3 (537.8–610.8)	850.1 (760–940.2)	778.2 (775.7–780.8)	1,044.7 (769.5–1,267.9)	1,050.9 (725.8–1,279.5)	ND
Infused volume (mL)	1,241.1 (1,000.0–1,500.0)	791.6 (750.0–833.3)	1,090.5 (1,000.0–1,181.1)	990.1 (986.8–993.4)	1,298.8 (1,000.0–1,500.0)	1,287.2 (967.7–1,500.0)	1,492.6 (1,485.2–1,500.0)
Energy (kcal /mL)^&^	1.23 (1.0–1.5)	1.9 (1.8–2.0)	1.26 (1.0–1.5)	1.51 (1.51–1.52)	1.21 (1.0–1.5)	1.20 (1.0–1.55)	1.05 (1.0–1.01)
Protein (g)	89.6 (60.0–139.5)	67.9 (67.5–68.2)	87.0 (78.5–100.0)	61.9 (61.7–62.1)	69.7 (58.3–82.5)	73.2 (55.9–138.0)	82.8 (82.5–83.2)
Carbohydrates (g)	161.5 (109.5–202.7)	130.8 (122.8–138.8)	170.8 (116.0–216.5)	104.8 (104.6–105.0)	140.9 (111.6–169.5)	194.8 (114.0–285.0)	189.5 (180–199.1)
Fiber (g)	4.9 (0.00–20.4)	5.3 (0.00–10.5)	8.8 (0.0–15.0)	NA	22.4 (13.5–36.0)	7 (0.0–28.7)	NA
Fat (g)	55.2 (39.3–69.0)	78.2 (75.0–81.4)	49.1 (30.2–69.0)	92.6 (92.5–92.7)	68.9 (58.5–81.6)	46.7 (11.2–65.1)	45.8 (41.6–49.9)
MCT (g)^#^	28.2 (15.0–42.0)	NA	19.0 (15.0–23.0)	12.1 (7.3–16.9)	10.4 (8.8–12.0)	25.3 (7.2–42.0)	12.0 (9.1–15.0)
SFA, g	23.5 (14.4–37.2)	ND	27.2 (21.5–33.0)	ND	9.9 (5.2–18.2)	22.4 (2.4–39.0)	22.6 (21.4–23.8)
MUFA, g	16.1 (5.1–27.6)	ND	16.4 (15–17.7)	ND	42.8 (27.7–55.2)	13.1 (2.7–42.4)	13.2 (8.8–17.7)
PUFA, g	5.8 (1.8–9.9)	ND	14.9 (10.8–19.0)	ND	15.4 (9.9–19.9)	4.7 (0.9–15.3)	4.8 (3.1–6.4)
PUFA/SFA ratio^†^	0.6 (0.2–1.2)	ND	0.5 (0.5–0.6)	ND	1.8 (0.8–3.0)	0.6 (0.2–2.5)	0.4 (0.4–0.5)
(PUFA+MUFA)/SFA^††^	1.8 (0.3–3.8)	ND	1.3 (0.9–1.7)	ND	7.1 (2.8–13.3)	1.6 (0.3–5.8)	1.0 (0.7–1.3)
Omega-3 (g)^*^	4.1 (0.9–6.7)	NA	NA	NA	3.7 (1.5–5.6)	2.6 (0.9–6.8)	-
Omega-6 (g)^**^	9.9 (9.9–10.0)	NA	NA	NA	10.3 (9.4–11.3)	8.4 (5.7–13.0)	-
EPA+DHA (mg)^***^	2,974.9 (368.8–4,900.0)	NA	3,809.1 (600–7,677.2)	NA	1,145.0 (409.6–2,550.0)	1,373.2 (507.0–3,552.0)	3,802.0 (3150.0–4,455.4)
L-Carnitine (mg)^*^	232.0 (96.0–1,050.0)	209.4 (198.0–220.8)	584.1 (118.1–1,050.0)	118.8 (118.4–119.2)	234.7 (117.0–560.7)	161.0 (100.5–300.0)	-
Arginine (g)^**^	10.4 (4.8–18.7)	NA	-	NA	NA	NA	16.3 (13.3–19.3)
Nucleotides (g)^***^	12.5 (0.3–18)	NA	-	NA	NA	NA	13.5 (0.3–26.8)

a *Results are expressed as mean, minimum (min), and maximum (max) per 1,500 kcal of infused enteral formula. (-), only in one of the group's formulae; NA, not in formula; ND, not declared*.

*MCT, medium-chain triglycerides; MUFA, monounsaturated fatty acid; PUFA, polyunsaturated fatty acid; SFA, saturated fatty acid*.

& *Energy content is expressed in kcal per ml of disease-specific formula as mean, minimum, and maximum*.

# *Mean MCT content according to the different therapeutic groups: metabolic stress (n = 8 formulae), cancer (n = 2 formulae), pulmonary (n = 2 formulae), diabetes (n = 2 formulae), malabsorption (n = 16 formulae), and surgery (n = 2 formulae)*.

† *PUFA/SFA ratio is composed of the polyunsaturated fatty acid (PUFA) content divided by the monounsaturated fatty acid (MUFA) content, both expressed in grams*.

†† *(PUFA+MUFA)/SFA ratio is the result of adding the content in grams of PUFA plus MUFA and dividing it by the content in grams of SFA*.

* *Omega-3 according to the different therapeutic groups: metabolic stress (n = 5 formulae), diabetes (n = 3 formulae), malabsorption (n = 7 formulae), and surgery (n = 1 formula)*.

** *Omega-6 according to the different therapeutic groups: metabolic stress (n = 2 formulae), diabetes (n = 3 formulae), malabsorption (n = 4 formulae), and surgery (n = 1 formula)*.

*** *EPA+DHA index is the sum of the eicosapentaenoic acid (EPA) content of the formula plus docosahexaenoic acid (DHA) expressed in mg per total volume of infused enteral formula. EPA+DHA mean content according to the different therapeutic groups: metabolic stress (n = 5 formulae), cancer (n = 3 formulae), diabetes (n = 9), malabsorption (n = 6 formulae), and surgery (n = 2 formulae)*.

^*^Mean carnitine content according to the different therapeutic groups: metabolic stress (n = 10 formulae), renal (n = 2 formulae), cancer (n = 2 formulae), pulmonary (n = 2 formulae), diabetes (n = 8 formulae), malabsorption (n = 8 formulae), and surgery (n = 1 formula).

^**^Mean arginine content according to the different therapeutic groups: metabolic stress (n = 4 formulae), cancer (n = 1 formula), and surgery (n = 2 formulae).

^***^Mean nucleotides content according to the different therapeutic groups: metabolic stress (n = 4 formulae), cancer (n = 1 formula), and surgery (n = 2 formulae).

**Figure 4:**
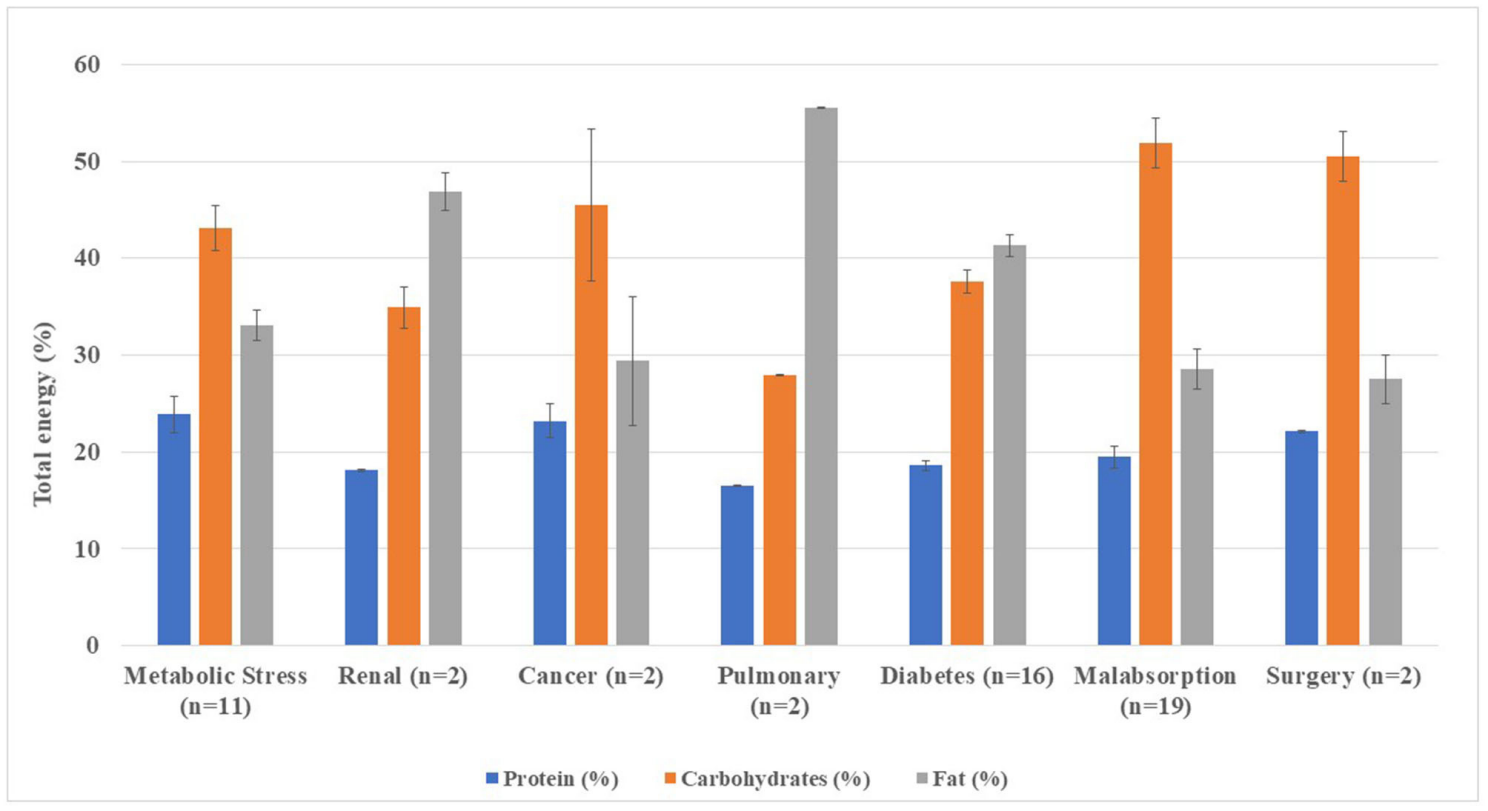
Macronutrient distribution in disease-specific enteral formula per 1,500 kcal infused.

DSFs for metabolic stress, diabetes mellitus, malabsorption, and surgery tended to be normocaloric (≤ 1.2 kcal/ml), whereas renal, pulmonary, and cancer formulae were mainly hypercaloric. Figure 4 shows macronutrient distribution of DSFs from 1,500 kcal.

Mean protein contents ranged from 16.5% (16.4%−16.6%) in pulmonary formulae (*n* = 2) to 23.9% (16.0%−37.2%) of TE in metabolic stress adapted formulae (*n* = 11), the latter with similar contents to the hyperproteic formulae previously assessed (G3, G4).

Mean carbohydrate percentages were higher among malabsorption (*n* = 19) with 51.9% (30.4–76.0%) and surgery formulae (*n* = 2) with 50.5% (48.0–53.1%) of TE. In turn, pulmonary formulae (*n* = 2) showed the lowest carbohydrate contents (27.9–28.0%). A 49% (*n* = 27) of DSFs for metabolic stress (*n* = 2), cancer (*n* = 2), renal (*n* = 1) and pulmonary (*n* = 1) diseases, and malabsorptive states (*n* = 5) had dietary fiber added. All diabetic formulae studied (*n* = 16) were fiber-enriched, while none of the surgical formulae.

**Figure 5:**
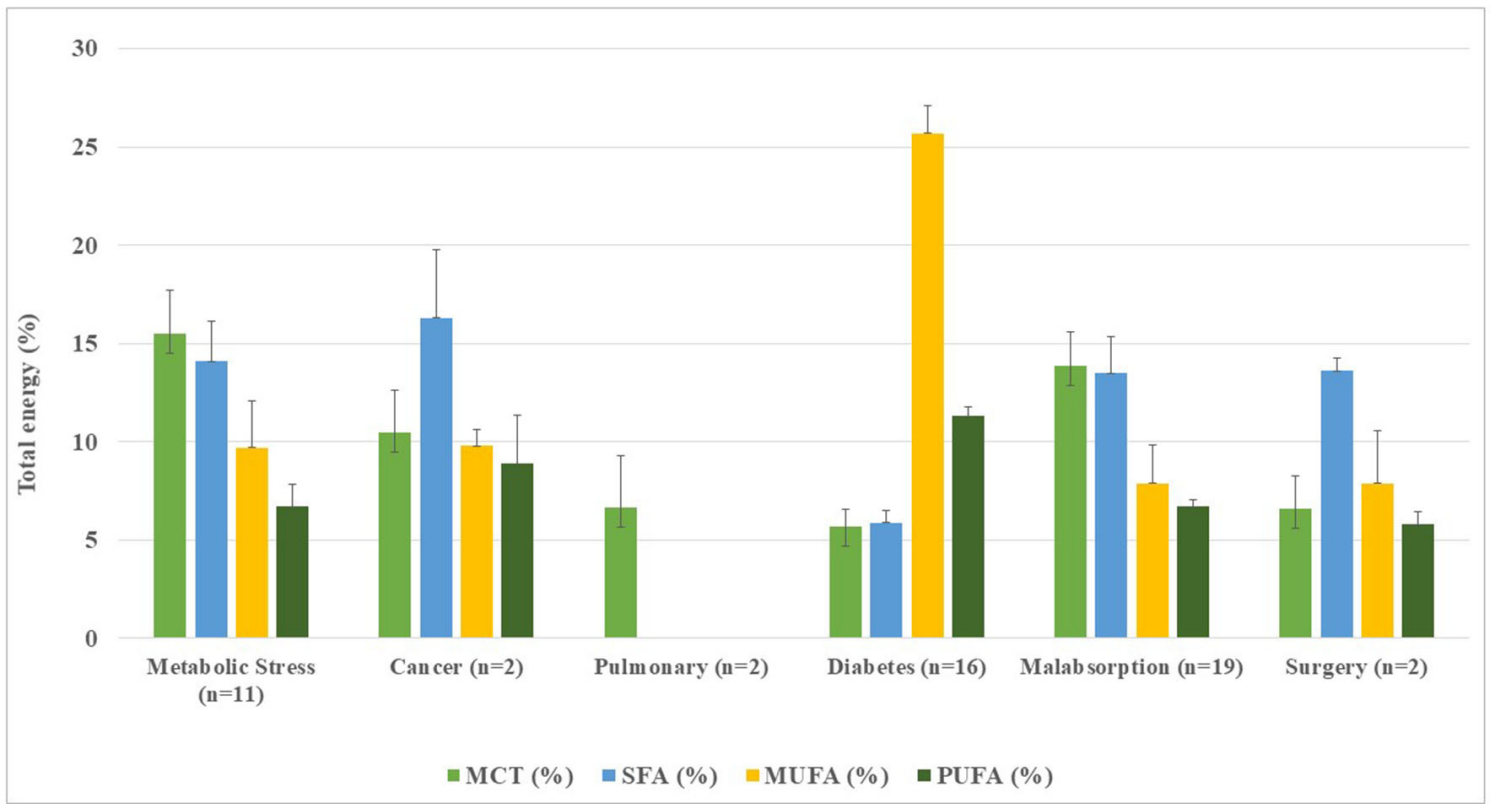
Distribution of fatty acids and medium-chain triglycerides in disease-specific enteral formulae per 1,500 kcal infused.

Mean fat contents were higher in pulmonary (*n* = 2) (r: 55.5–55.6%) and renal formulae (*n* = 2) (r: 45.0–48.8%). The lowest mean fat content was observed among surgery 27.5% (r: 24.9–29.9%) and malabsorption formulae 28.6% (r: 6.7–39.1%). The MCTs were found in 58.2% (*n* = 30) of the DSFs, mainly in malabsorptive (29%; *n* = 16) and metabolic stress (14.5%; *n* = 8) conditions. All oncology, pulmonary, and surgical formulations had MCTs, while the renal formula had no added MCTs (Table 2, Figure 5).

Fatty acid distribution of DSFs (Table 2, Figure 5), mean SFA contents ranged from 13.5% (1.4–23.4%) in malabsorption formulae (*n* = 19) to 16.3% (12.9–19.8%) of TE in cancer-oriented formulae, exceeding current CV prevention recommendations (14). Except in the diabetes formulae (*n* = 16), where SFA accounted for 5.9%, a higher proportion of SFA vs. MUFA was found, showing a clearly unbalanced lipid profile. MUFA contents ranged from 7.9% (1.6–25.5%) in malabsorption formulae (*n* = 19) to 9.8% (9.0–10.6%) of TE in cancer formulae (*n* = 3). Noteworthy, contents of MUFA from diabetic formulae (*n* = 16) accounted for 25.7% (16.6–33.1%). The PUFA frequency ranged from 5.8% (5.2–6.5%) in surgery formulae (*n* = 2) to 11.3% (6.3–20.7%) in diabetes formulae (*n* = 16). There was no available fatty acid data declared to date for renal and pulmonary formulae.

The PUFA/SFA ratio ≥ 0.5 was found in 54.9% (*n* = 28). Cancer and diabetes formulae met the PUFA/SFA target ratio ≥ 0.5, while a lower proportion of this ratio for malabsorption (11.8%; *n* = 6), metabolic stress (5.9%; *n* = 3), and surgery (2%; *n* = 1) was found (data not shown). The mean (PUFA+MUFA)/SFA ratio was <2.0 in all groups, except for the diabetic's formulae (*n* = 16) whose ratio was 7.1 (r: 2.8–13.3) in line with their higher MUFA and PUFA contents.

Of the DSFs, 45.5% (*n* = 25) had added EPA and DHA, found in diabetic (17.6%), malabsorption (11.7%), and metabolic stress formulations (9.8%). All of the cancer and surgery formulations had EPA and DHA added, but none for the renal or pulmonary diseases (Table 2). l-carnitine was 60% of the DSFs, especially in the metabolic stress (18.2%), diabetes, and malabsorption (29.1%) formulae. Formulations for cancer, renal, and lung diseases contained l-carnitine, with the highest content in cancer formulae. Arginine and nucleotides were found only 12.7%, mainly in metabolic stress (*n* = 4), cancer (*n* = 1), and surgery (*n* = 1) formulae.

## Discussion

The results of this study show, for the first time, the macronutrient composition of selected FSMPs, their fatty acid profile and fat quality indices, and the content of some immune-modulatory nutrients in enteral formulae on the European market.

DRM costs billions per year worldwide (1, 17) justifying the need for a nutritional approach based on the analysis of nutritional composition in enteral formulae. The selection of enteral formulae usually depends on the severity of the underlying disease and the metabolic stress that accompanies DRM as well as the patient's clinical condition (5, 6, 18). Results from this study showed a higher proportion of SFs vs. DSFs, being in both, mostly polymeric formulae. In particular, peptide-based oligomeric formulae were found for metabolic stress and malabsorptive states, representing only 18% of the enteral products evaluated. European clinical guidelines (19–21) recommends the use of peptide-based oligomeric formulae in patients with acute pancreatitis, intestinal absorption severely impaired, or those in whom use of polymeric SFs cannot be tolerated. SFs with whole proteins (fiber added or fiber-free), considering the nutritional requirements of the underlying disease, are recommended as the first-choice nutritional intervention in the majority of disease states (19–30). Thus, the study findings showed that SFs and DSFs polymeric normo-hyperproteic with different energy density contributed to at least two-thirds of the enteral formulae assessed. In fact, standard polymeric formulae have showed broad versatility, good tolerance, and lower healthcare costs in up to 95% of patients with normal gastrointestinal functionality for digestion and absorption of nutrients (7), justifying their wide distribution in the European market. Additionally, DSFs with intact, partially, or fully hydrolysed proteins are used whenever they show additional benefits over the administration of SFs (e.g., poor tolerance to SFs) and/or when certain metabolic disorders (e.g., hyperglycemia, electrolyte imbalance, and malabsorption) are not well controlled due to the disease states (19, 28, 29, 31).

Macronutrient distribution in FSMPs can vary from the origin and source of the nutrient in chemical form, biological value (protein), digestibility, fermentability (carbohydrate), and nutrient metabolic utilization (32). Mean protein contents ranged from 16.2 to 22.9% in normo-hyperproteic SFs, whereas higher protein contents were found for metabolic stress, cancer, surgery, and malabsorptive states. In catabolic states, high protein formulae are useful in the support of selected metabolically stressed patients and those with moderate-severe comprised nutritional status (21, 22, 24, 25, 27, 33). In fact, it also highlighted the quality of the protein in the formula (essential and non-essential amino acids) and the origin of the protein (vegetable vs. animal or a mixture of both). High-quality protein derived from animal sources (e.g., whey isolate/concentrate and egg origin) has high biological value and net protein utilization (NPU) ranging from 92 to 94%, while a lower biological value is from vegetable and/or soy isolate/concentrate protein (NPU: 61–61.4%) (34). Of note, whey proteins contain all essential and non-essential amino acids and are enriched in branched chain amino acids, in particular leucine, a key amino acid for metabolic protein synthesis (35). Since the nutritional recovery depends in part on the selection of enteral formula, a key point is to assess the content and origin of the proteins administered by tube feeding.

Carbohydrates represent the largest energy source in enteral formulae and come from maltodextrin and varying amounts of corn syrup, fructo-oligosaccharides (FOS) as fructans, galacto-oligosaccharides (raffinose), fructose, inulin, and polyols (maltitol) (32, 36). Results from this study showed that lower carbohydrate contents were found in hypercaloric formulae. Noteworthy, carbohydrate content was <50% for pulmonary, renal, diabetes, and metabolic stress formulae. In addition, 46% of the DSFs had soluble fiber from non-starch polysaccharides (inulin, guar gum, oats, and FOS), while insoluble fiber came from resistant starch and lignin. Notably, enteral formulae enriched with a mixture of soluble and insoluble fiber can help normalize bowel function, which is often compromised during illness (23, 37, 38). In a long-term enteral tube feeding study (39), fiber-enriched SFs were shown to increase stool short chain fatty acids and total number of gut bacteria with beneficial effects on host health, findings that may contribute to improved bowel function. Conversely, fermentable fibers (monosaccharides, oligosaccharides, disaccharides, and polyalcohols) may also cause intolerance or diarrhea in patients with DRM (36). However, the evidence available to date is limited, and further studies are needed to assess the effects of fiber-enriched enteral formulae on gastrointestinal function, their impact on gut microbiota, and potential host immune-enhancing.

Fat content in enteral formulae should be considered based on the energy, fatty acid, and omega-6 and omega-3 contents. Analysis of the data showed that the amounts of fat are higher in the G2 and G4 (r: 33.8–35%), while the renal, pulmonary, and diabetic formulations had even higher fat content (≥41%). In fact, the higher fat content in some formulae are based on carbon dioxide reduction (pulmonary formula), imposed volume restrictions (renal formula), and optimized glycemic control with a healthy fat profile in diabetics. However, according to the current clinical nutrition guidelines (22, 28, 29, 31), the default indication should be based on the prescription of a standard polymeric formulation in the preceding diseases.

Overall, the origin of the fat in the SFs was from LCT, while two-thirds were also mixed with MCT, being mean values slightly superior in the high-protein SFs. Likewise, MCT was mainly added in 32 formulations being in DSFs broadly represented. Moreover, results from this study showed that 30% of SFs and 60% of DSFs were enriched with l-carnitine, specifically in cancer and diabetes formulae. However, at present, European clinical guidelines on nutrition do not support the inclusion of l-carnitine with such effects in enteral formulae.

Considering the results of the fatty acid profile and the guidelines for guiding CVD prevention (14), normoproteic formulae, both normocaloric and hypercaloric (G1, G2) exceeded the recommended SFA percentage (>10%). Of the DSFs with reported SFA data, 46.5% of formulae exceeded the recommended SFA. In contrast, only 7 SFs and 12 diabetes-specific formulations met the CVD recommendations for MUFA and PUFA. Results from PUFA/SFA ratio (≥0.5) and PUFA+MUFA-to-SFA ratio (>2.0) showed that most SFs (80.4 and 85.7%, respectively) had a healthy fatty acid profile. The highest MUFA content was in diabetic formulae, whereas fatty acid profiles were not declared by the manufacturer in renal and pulmonary formulae. European expert group (31) endorses the utilization of diabetes-specific formulae for nutritional support of obese and diabetics patients. Recent studies (40, 41) supported the use of high MUFA formulae in patients in critical care settings with or without diabetes. Therefore, given the impact of fat content on CV health and mortality, it is useful for health professionals to have at their disposal up-to-date information on the fatty acid profile of enteral formulae.

A wide variability in the immuno-modulatory substrates such as omega-3, omega-6, EPA, DHA, arginine, and nucleotides were reported. In this study, omega-3, omega-6, and/or EPA and DHA contents were in similar proportions in SFs and DSFs. Selected standard formulae (G1, G2) with different energy content and DSFs for diabetes, metabolic stress, malabsorptive, and surgery conditions were enriched with both omega-3 and EPA+DHA. Notably, SFs (G4), pulmonary, and renal formulae did not contain omega-3, omega-6, and/or EPA+DHA. In addition, only polymeric DSFs for metabolic stress, cancer, and surgery were enriched in arginine and nucleotides. Moderate evidence is in favor of immune-modulatory formulae for its potentially immune-enhancing properties. *Guidelines on Nutrition* (42) recognizes some health benefits of enriched omega-3 and considers the recommendations that the normal diet should contain a daily intake of 500 mg of EPA and DHA (43). However, at present, immune-modulatory formulae are only recommended for patients with upper gastrointestinal tract cancer undergoing surgical resection as part of routine perioperative care (44, 45) and major cancer surgery (27).

Some potential strengths and limitations must be highlighted in this study. To the best of our knowledge, this is the first descriptive study assessing the nutritional composition of selected European enteral formulae. The above findings may guide health professionals in making informed decisions about the use of enteral formulae. However, some potential limitations should be considered. Our results should be interpreted taking into account the study design and the nutritional information declared in the data sheet of FSMPs from manufacturers. Currently, protein, lipid, and carbohydrate sources are not fully declared in the manufacturers' formulations or websites. This information is relevant and necessary for nutritional intervention as it helps the professional to know the composition of certain nutrients (e.g., FODMAP and fatty acids) in the selection of enteral formulas in disease conditions. Likewise, knowing the source and origin of nutrients allows the evaluation and/or reformulation of some of the available formulas. Basically, our results are limited by the fact that, at present, there are no similar studies published with which our findings can be compared. Considering those premises, we emphasize the need for further studies assessing FSMPs to a best decision-making in the selection of enteral formulae.

In conclusion, a higher proportion of SFs were found, in line with current European guidelines on clinical nutrition. Results of this study may help to evaluate the nutritional value and explore its potential usage in disease prevention and nutritional intervention. As the improvement of healthcare through the delivery of optimal nutrition contributes to the nutritional recovery of patients and to the efficiency and sustainability of healthcare systems, further studies evaluating nutritional composition and long-term effects of different EFs are needed.

## Data Availability Statement

The original contributions presented in the study are included in the article/Supplementary Material, further inquiries can be directed to the corresponding author/s.

## Author Contributions

MR performed the data collection, statistical analyses, data discussion, and writing and intellectual discussion of the manuscript. AM-B, TP, and AP participated in the study revising the manuscript. GV-M provided intellectual content of critical importance to the work described. MS-V reviewed data collection, statistical analyses, data discussion, and writing and intellectual discussion of the manuscript. All authors contributed to the article and approved the submitted version.

## Conflict of Interest

The authors declare that the research was conducted in the absence of any commercial or financial relationships that could be construed as a potential conflict of interest.

## Publisher's Note

All claims expressed in this article are solely those of the authors and do not necessarily represent those of their affiliated organizations, or those of the publisher, the editors and the reviewers. Any product that may be evaluated in this article, or claim that may be made by its manufacturer, is not guaranteed or endorsed by the publisher.
