# Supplementary material for: A Descriptive Analysis of Macronutrient, Fatty Acid Profile, and Some Immunomodulatory Nutrients in Standard and Disease-Specific Enteral Formulae in Europe
Source: Front Nutr. 2022 May 10;9:877875. doi: 10.3389/fnut.2022.877875 (PMC9129913; doi:10.3389/fnut.2022.877875)
Supplement: Supplementary file 1 [file Data_Sheet_1.PDF]

Supplementary material. Annex 1. Analysed enteral formulae

The enteral formulae analysed are listed in alphabetical order according to their trade name.

Atempero enteral
Bi1 ViaDiacare
Bi1 ViaDiacare hp/hc
Bi1 Viafibra
Bi1 Via hp/hc
Bi1 ViaProcare
Diaba
Diaba Plus
Diben
Diben 1.5 HP
Dienat G Enteral
Dietgrif diabetic
Dietgrif Energy
Dietgrif Polipeptidic
Dietgrif standard
Dietgrif standard fibre
Dietgrif standard high protein
Dietgrif TCM
Ensure HN
Ensure Plus Advanced RTH
Ensure Plus HN
Fresubin 2 kcal HP
Fresubin 2 kcal HPFibre
Fresubin Energy
Fresubin HP Energy
Fresubin HP Energy Fibre
Fresubin Intensive
Fresubin Original
Fresubin Original Fibre
Glucerna 1.0
Glucerna 1.2
Glucerna 1.5
Glucerna Select
Impact Enteral
Impact Peptide 1.5
Isosource 1.0 HP
Isosource 1.2
Isosource 1.5
Isosource 2.0
Isosource Energy
Isosource Energy Fibre
Isosource Fibre 1.2
Isosource Mix
Isosource Protein
Isosource Protein Fibre
Isosource Standard
Isosource Standard Fibre
Jevity HiCal
Jevity Plus
Jevity Plus HP
Jevity RTH
Nepro HP Enteral
Novasorce GI Protein
Novasource Diabet
Novasource DiabetPlus
Novasource GI Advance
Novasource GI Balance
Novasource GI Control
Novasource GI Forte
Novasource Renal
Nutrison
Nutrison Advanced Diason
Nutrison Advanced Diason HP
Nutrison Advanced Peptisorb
Nutrison Concentrated
Nutrison Energy
Nutrison Energy Multifibre
Nutrison MCT
Nutrison Multifibre
Nutrison PeptisorbPlus HEHP
Nutrison Protein Advance
Nutrison Protein Intense
Nutrison ProteinPlus
Nutrison ProteinPlus Energy
Nutrison Proteinplus Multifibre
Nutrison Soya
Nutrison Soya Multifibre
Nutrison1000 Complet MultiFibre
Nutrison1200 Complet MultiFibre
Nutavant Fibre
Nutavant Standard
Osmolite
Osmolite HNPlus
Oxepa
Peptamen 1.5
Peptamen AF 1.2
Peptamen AF Enteral
Peptamen Enteral Pepta 1.0
Peptamen HN Neutral Dual
Peptamen Intense
Peptamen Intense VHP
Peptamen Prebio
Perative RTH
Promote
Prosure
Pulmocare
Replete
Supportan Easybag
Survimed OPD
Survimed OPD-HN
Sondavant HP Fibre
Sondavant Plus
Sondavant Plus Diabet
Sondavant Standard
TwoCal
T-Diet Standard
T-Diet Energy
T-Diet hp
T-Diet hp not fibre
VitalPeptido1.5
Vivonex RTF
